# Modeling the role of the close-range effect and environmental variables in the occurrence and spread of Phragmites australis in four sites on the Finnish coast of the Gulf of Finland and the Archipelago Sea

**DOI:** 10.1002/ece3.986

**Published:** 2014-02-28

**Authors:** Anas Altartouri, Leena Nurminen, Ari Jolma

**Affiliations:** 1 Department of Civil and Environmental Engineering, School of Engineering, Aalto UniversityP.O. Box 11000, FI-00076, AALTO, Espoo, Finland; 2 Department of Environmental Sciences, University of HelsinkiP.O. Box 65, FI-00014, Helsinki, Finland

**Keywords:** Baltic Sea, boosted regression trees, common reed, habitat suitability, machine learning, species distribution models

## Abstract

*Phragmites australis*, a native helophyte in coastal areas of the Baltic Sea, has significantly spread on the Finnish coast in the last decades raising ecological questions and social interest and concern due to the important role it plays in the ecosystem dynamics of shallow coastal areas. Despite its important implications on the planning and management of the area, predictive modeling of *Phragmites* distribution is not well studied. We examined the prevalence and progression of *Phragmites* in four sites along the Southern Finnish coast in multiple time frames in relation to a number of predictors. We also analyzed patterns of neighborhood effect on the expansion and disappearance of *Phragmites* in a cellular data model. We developed boosted regression trees models to predict *Phragmites* occurrences and produce maps of habitat suitability. Various *Phragmites* spread figures were observed in different areas and time periods, with a minimum annual expansion rate of 1% and a maximum of 8%. The water depth, shore openness, and proximity to river mouths were found influential in *Phragmites* distribution. The neighborhood configuration partially explained the dynamics of *Phragmites* colonies. The boosted regression trees method was successfully used to interpolate and extrapolate *Phragmites* distributions in the study sites highlighting its potential for assessing habitat suitability for *Phragmites* along the Finnish coast. Our findings are useful for a number of applications. With variables easily available, delineation of areas susceptible for *Phragmites* colonization allows early management plans to be made. Given the influence of reed beds on the littoral species and ecosystem, these results can be useful for the ecological studies of coastal areas. We provide estimates of habitat suitability and quantification of *Phragmites* expansion in a form suitable for dynamic modeling, which would be useful for predicting future *Phragmites* distribution under different scenarios of land cover change and *Phragmites* spatial configuration.

## Introduction

The common reed *Phragmites australis* is a perennial vascular plant found in wetlands with a nearly worldwide distribution (Haslam 1972; Lambertini et al. 2008). In North America, the common reed is considered a highly problematic cryptic invader because the Eurasian haplotype as a strong competitor is reported to override the native American haplotype (Saltonstall 2002, 2003) by, *for example,* a more aggressive shoot initiation from rhizomes and higher salinity tolerance than the native haplotype (Vasquez et al. 2005). In central Europe, on the other hand, the native reed beds have undergone significant deterioration known as the reed dieback syndrome (*e.g.,* Koppitz 1999). This reed decline has been associated with excess eutrophication leading to deterioration of the rhizomes (Ostendorp 1989) and decline of genetic diversity of reed beds (Koppitz et al. 1997). On the coastal areas of the Baltic Sea, *Phragmites australis* is a native wide-spread helophyte playing an important role in the ecosystem dynamics of shallow coastal areas (Meriste et al. 2012).

In the Baltic Sea, the reed beds, situated at the land–water interface, protect the shoreline from wave-induced bank erosion, mitigate sediment-borne internal nutrient loading, and act as buffers for catchment-borne external loading (Kaitaranta et al. 2013). *Phragmites* distribution is also a corner stone in coastal ecology, as reed belts sustain high biodiversity by providing nesting areas for birds (Huhta 2009; Meriste et al. 2012) and spawning areas for fish (Härmä et al. 2008; Lappalainen et al. 2008). The role of *Phragmites* on the functioning of coastal areas is, however, contradictory, as during the last decades, *Phragmites* has spread along the shore areas of the Baltic Sea and is claimed to also have negative feedbacks on coastal ecosystems due to its rapid spread (Huhta 2009; Pitkänen et al. 2013). *Phragmites* is a strong competitor and once established in an area often outcompetes and shades other plant species decreasing local biodiversity (Munsterhjelm 1997). In the coastal area of the Gulf of Finland (GOF) and the Archipelago Sea, the increase in *Phragmites* distribution during the last few decades has been associated with multiple changes in human activities (Ojala and Louekari 2002) and has raised the interest and concern of local people (IBAM 2011; Lampén 2012).

A number of studies have examined the causes of *Phragmites* intensive expansion in different regions. As a worldwide common factor, human disturbance in coastal areas has been found to facilitate *Phragmites* dispersal (Burdick and Konisky 2003; Silliman and Bertness 2004; Bart et al. 2006; King et al. 2007; Chambers et al. 2008). There is evidence of pronounced *Phragmites* prevalence on shorelines adjacent to urbanized (King et al. 2007) and agricultural land (Chambers et al. 2008). Maheu-Giroux and De Blois (2007) presented point pattern analysis of *Phragmites* expansion in linear wetlands. They found that *Phragmites* expanded with higher rates in linear anthropogenic habitats compared with natural wetlands. Additionally, *Phragmites* is a pioneer species, being among the first species to settle on virgin soil after mechanical alterations of land, such as dredging and near-shore building (Pitkänen et al. 2013). Along the Baltic coast, the increasing eutrophication due to excess nutrient runoff from land together with decreased grazing pressure has led to the spread of large perennial species such as *Phragmites* (Jutila 2001; von Numers 2011; Pitkänen et al. 2013). In the Finnish Archipelago, the expansion of *Phragmites* has been rapid in soft and sheltered areas where reed belts have become denser and wider (Pitkänen et al. 2013). *Phragmites* is also witnessed to expand outward in new areas in the archipelago (von Numers 2011). This suggests the establishment of new suitable habitats through organic matter settlement to formerly soil-poor outer archipelago areas. It also reflects the absence of the spread controlling role of grazing (Jutila 2001; von Numers 2011) as annual cutting of reed beds by cattle controlled the vegetative growth and conceivably the probability of seed formation of *Phragmites*.

*Phragmites* is known to spread both generatively through seed formation and seedling growth and vegetatively by rhizome growth of clones (Koppitz 1999; Belzile et al. 2010). Seed production may be abundant and occurs in the fall, and seeds can be dispersed by wind during ice cover or through wave transportation during ice-free time (Baldwin et al. 2010), with dispersal distance up to 10 km (Fér and Hroudová 2009). The seeds of *Phragmites* can float for several days (Fér and Hroudová 2009), and germination time is one year (Baldwin et al. 2010). Sexual dispersal through seed settlement can occur in new suitable growing sites at the shoreline with optimum sediment property and moisture and space free of vegetation. This is known as the settlement phase (Koppitz and Kühl 2000) after which the seedlings propagate vegetatively to occupy the free niches, a phase known as the propagation stage. During the last stationary phase, the various genotypes compete for space and the best-adapted clones to the site prevail (Koppitz and Kühl 2000). Therefore, the genetic diversity in old reed bed is quite low, and the stands consist of only few best-adapted vegetatively dispersing clones (Koppitz et al. 1997). As seed germination and seedling growth cannot occur in submerged conditions (Weisner and Ekstam 1993; Weisner et al. 1993) or under heavy shading and competition for space, usually local close-range spread of *Phragmites* beds is due to vegetative horizontal growth of rhizomes. Vegetative growth rate can vary from 1 to 4 m yearly (Weisner 1987; Clevering and Van der Toorn 2000). In the coastal areas of Finland, new areas of long-distance colonization can be inhabited by seed dispersal and seedling establishment, or alternatively vegetatively through rhizome bits cut out of reed beds and transported by waves, but close-range colonization and dispersal occur mainly through clonal growth (Koppitz et al. 1997; Mal and Narine 2004; Fér and Hroudová 2009; Kettenring and Mock 2012).

Topographic factors can influence the occurrence of *Phragmites*. Having a stiff and strong stem, *Phragmites* is more resistant to wave exposure at shallow water than other helophytes (Coops and van der Velde 1996), which partly explains the ability to colonize new sites in the sea area. However, open shorelines prone to heavy surfs are unfavorable habitats for *Phragmites* (Coops and van der Velde 1996; von Numers 2011). Additionally, increasing water depth is a strong selective force in limiting reed dispersion because internal aeration pathways suffer as transportation of oxygen to the roots becomes more difficult when the plant grows deeper (Huhta 2009; Engloner and Major 2011). Water depth therefore regulates the seaward expansion (Meriste et al. 2012) as maximum growth depth of *Phragmites* in sheltered areas is ca. 2 m (Luther 1951; L. Nurminen, pers. obs.). Understanding the reasons behind and mechanisms of the spreading of *Phragmites* on coastal ecosystems of the Baltic Sea is of timely importance for modeling this phenomenon. Targeted management and planning of the area can greatly benefit from predictive modeling of *Phragmites* distribution.

Species distribution modeling/models (SDMs) are useful and widely applied tools in environmental conservation and management (Guisan and Zimmermann 2000; Austin 2002). A SDM attempts to spatially predict the occurrence or abundance of a species mostly by relating data on the species distribution with the environmental and topographic characteristics of associated locations (Elith and Leathwick 2009). Various approaches and methods are used for modeling species distributions, and new trends are emerging in SDM (Zimmermann et al. 2010). Novel methods, including machine learning (ML) techniques, are increasingly adopted in SDM and found to improve prediction capabilities (Elith et al. 2006; Hochachka et al. 2007; Elith and Graham 2009). Advances in algorithms and computation power facilitate the use of these methods and allow handling large data, both in the number of instances and predictors. Advancement, although to a lesser extent, has also occurred in different directions such as the use of dynamic models in studies of species distributions (Robinson et al. 2011).

A correlative approach to SDM utilizes data of species occurrences, environmental gradients, and topographic variables to delineate potentially suitable habitats and predict species occurrences in unsampled geographic locations. In spatial analysis, the effect of the geographic vicinity on a location's characteristics is well established (Tobler 1970). Significant parts of spatial processes are explained by the surrounding influence. The realization of this influence in geographic space causes a phenomenon known as autocorrelation. Legendre (1993) defines autocorrelation as “the property of random variables taking values, at pairs of locations a certain distance apart, that are more similar (positive autocorrelation) or less similar (negative autocorrelation) than expected for randomly associated pairs of observations.” Autocorrelation can occur in both space (SAC) and time. In ecology, autocorrelation is intrinsic to species distributions by means of dispersal (Wintle and Bardos 2006; Dormann 2007a). In species with close-range vegetative/clonal dispersal such as *Phragmites*, spatial dependency is more pronounced due to the vegetative expansion with rhizomes. Therefore, it is important for the study of *Phragmites* dynamics to consider the close-range neighborhood effect together with the influential environmental and topographic variables.

A number of coastal ecosystem studies of the Finnish coasts addressed the prevalence and expansion of *Phragmites* (*e.g.,* Ojala and Louekari 2002; Ikonen and Hagelberg 2007; Huhta 2009), its role in the ecosystem and interaction with other species (*e.g.,* Härmä et al. 2008; Lappalainen et al. 2008; Kaitaranta et al. 2013), and the social interest and concern that it raises (IBAM 2011; Lampén 2012). However, few studies have attempted to map *Phragmites* occurrences (Luther 1951; Suominen 1998; Pitkänen 2006), and yet fewer attempted to model its distribution in the area (von Numers 2011; Pitkänen et al. 2013). Suominen (1998) delineated *Phragmites* colonies for three sites in the Archipelago Sea from aerial photographs which date back to the second half of the last century. Pitkänen (2006) used satellite imagery to map reed colonies along the whole Finnish coast of the GOF, the Archipelago Sea, and areas Northeast Estonia. They reported that although *Phragmites* colonies were successfully mapped in some areas, the overall accuracy of the resultant map has significant error rates that prevent its use in other applications. Pitkänen et al. (2013) examined contemporary records of the occurrence of a number of species in comparison with historical data from 1930s and 1940s surveyed by Luther (1951). They observed significant increase in *Phragmites* occurrences in the contemporary survey records compared with the historical data. von Numers (2011) compared the occurrence of a number of species in historical and contemporary data and used logistic regression to test variables that exert influence on the occurrence and colonization of species. He found significant *Phragmites* prevalence at nonrocky and sheltered shores, although a shift in *Phragmites* occurrences toward less sheltered islands was observed.

Despite the contribution of these studies to the effort of understanding the dynamics of *Phragmites* spread on the Finnish coasts, a number of questions are still to be investigated. There is a need for quantifying endogenous and exogenous factors playing roles in the colonization of shores by *Phragmites*, as a basis for a predictive SDM. Compared to static mapping, a SDM utilizes input of *Phragmites* occurrences (provided, *e.g.,* by manual delineation or spectral analysis of aerial photographs or satellite imagery) to build a model capable of predicting distributions of *Phragmites* in unsampled geographic locations and/or time frames. Moreover, evaluation of various scenarios for the purpose of managing and planning of the coastal ecosystem becomes possible using a SDM. We present here an analysis of *Phragmites* occurrence and spread in four sites on the Finnish coast of the GOF and the Archipelago Sea. We examine environmental and topographic predictors of *Phragmites* occurrence and test spatial patterns of its dynamics. We adopt a cellular representation of the phenomenon and consider multiple scales in the analysis. Laying a foundation for a spatio-temporal model of *Phragmites* distribution, the objectives of this study were as follows: (i) to examine the occurrence of *Phragmites* in relation to a number of environmental predictors, (ii) to examine and quantify the effect of close-range dispersal on *Phragmites* expansion, and (iii) to develop a predictive model of *Phragmites* distribution and produce maps of habitat suitability.

## Materials and Methods

### Study area and data

**Figure 1:**
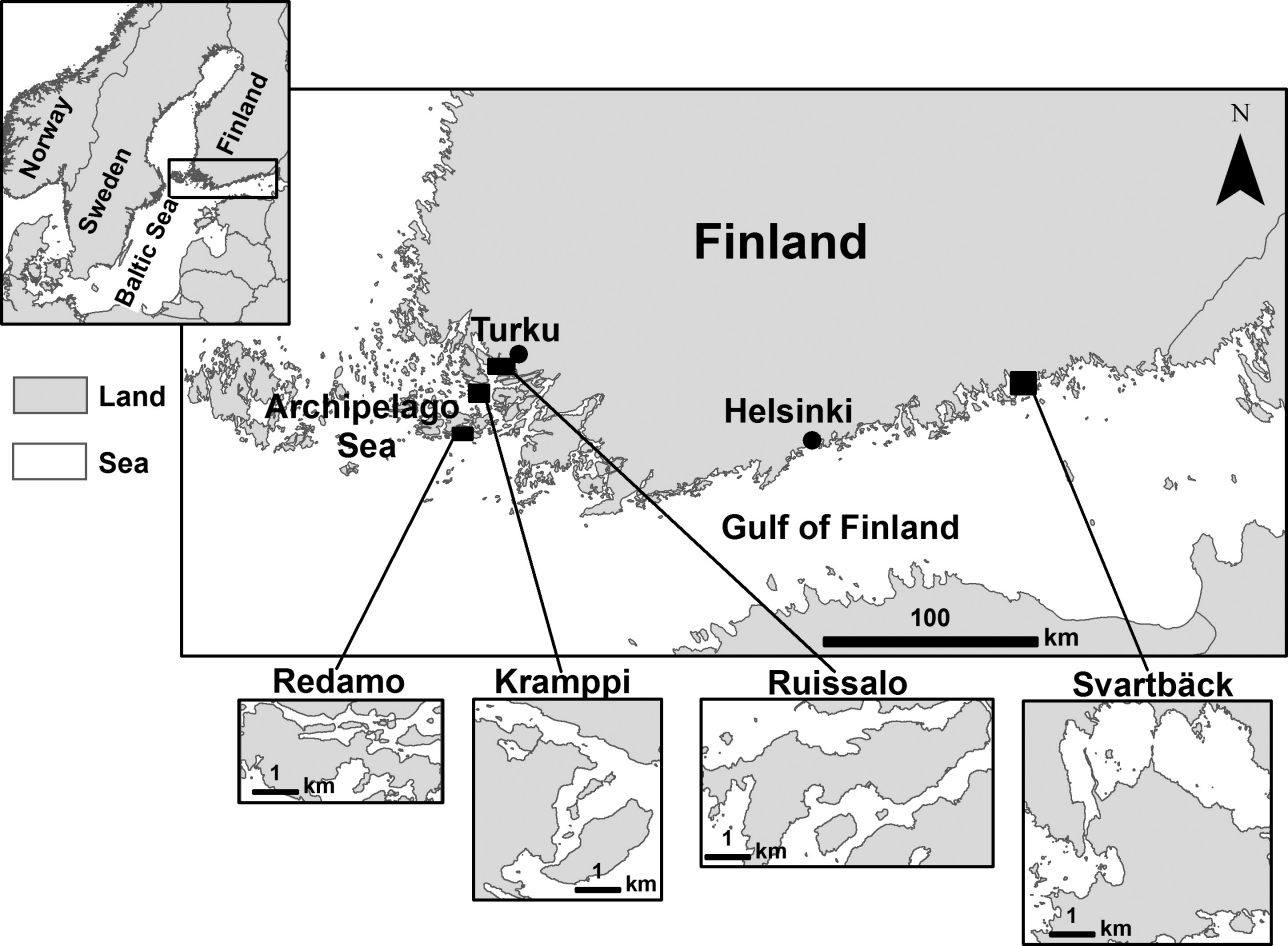
Location map of the study area.

Our study area consisted of four sites located in the Archipelago Sea and the Finnish coast of the GOF (Fig. 1). The area has witnessed significant *Phragmites* spread both seaward and into clear shores in the last few decades (Huhta 2009; von Numers 2011; Pitkänen et al. 2013). Sites located in the Archipelago Sea are near the city of Turku, at Ruissalo, Kramppi, and Redamo islands. The fourth site is in Svartbäck (Purola), on the Eastern part of the Finnish coast of the GOF, close to the outlet of River Kymijoki, which is one of the major rivers flowing into the GOF. In addition to data availability, the choice of the study sites took in consideration representation of different zones, such as the inner (Ruissalo), outer (Redamo), and intermediate (Kramppi) Archipelago as well as the Eastern part of the Finnish coast of the GOF (Svartbäck). These zones have varying coastal characteristics including shore openness, water quality, and shore development.

**Figure 2:**
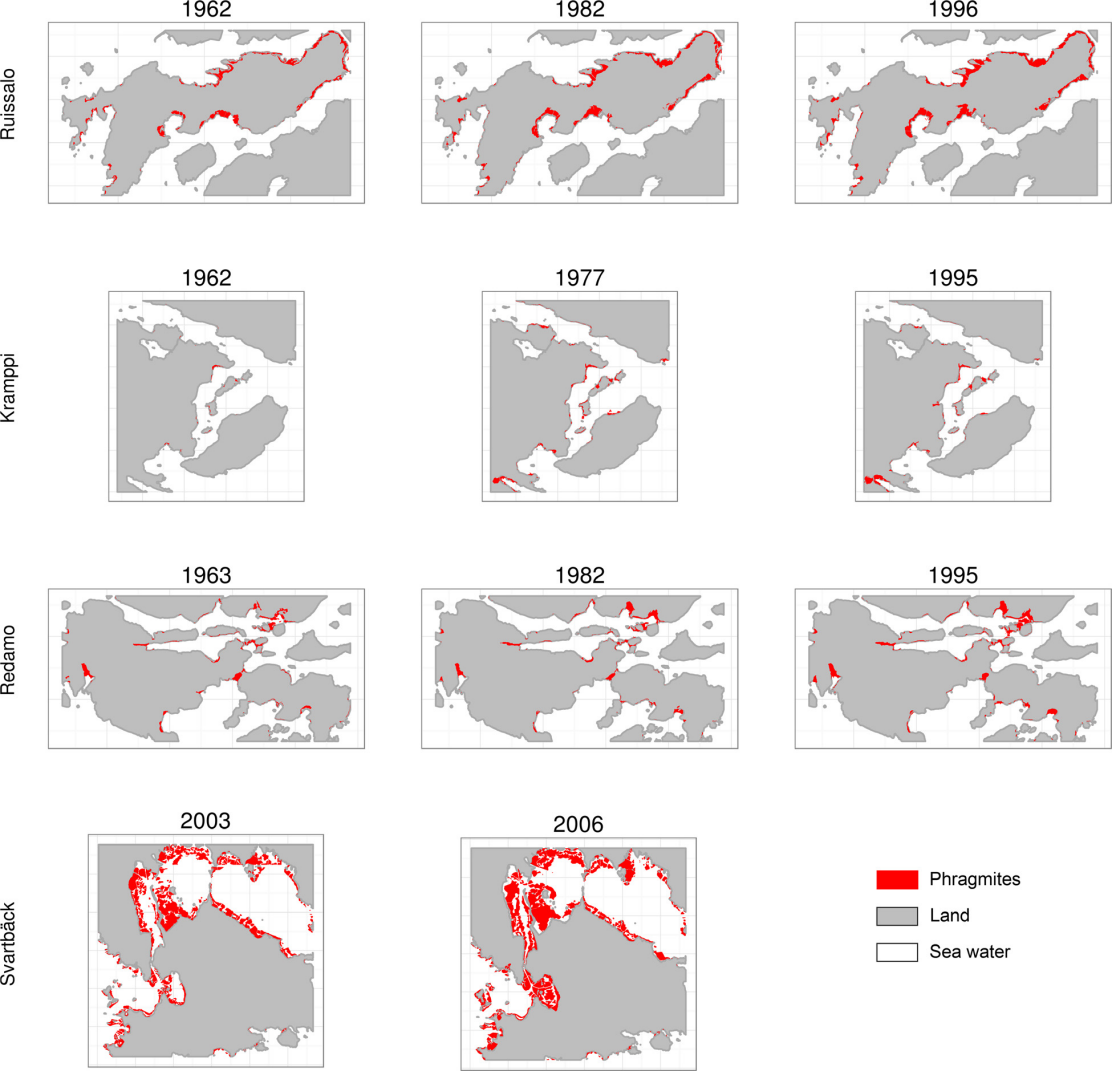
Observed *Phragmites* distributions in each study site in different years.

Maps of *Phragmites* distributions in different years for each site (Fig. 2) were provided by the Finnish Environment Institute (SYKE). Maps of *Phragmites* in the Archipelago sites were delineated by Suominen (1998) from aerial photographs that were taken in spring and early summer (April 24 to June 16). He introduced corrections on some of the delineations after a boat visit to the field in 1997. The smallest reed patches identified were about 25 m^2^ in area (Suominen 1998). Svartbäck *Phragmites* maps were delineated by experts in SYKE from aerial photographs taken in July 2003 and August 2006. Bathymetry grids with pixel resolution of 5 m were derived for all sites, using ANUDEM program (Hutchinson 1988, 1989) in ArcGIS 10 (ESRI 2011), from elevation and depth contour lines and depth measurement points in the topographic maps available from the National Land Survey of Finland. Grids of relative shore openness with pixel resolution of 10 m were derived from the abstraction of fetch lines, the stretch of water surface over which waves can develop freely (Lundqvist et al. 2006). In addition, grids of the Euclidean distance to the closest river outlet with pixel resolution of 5 m were calculated for each site. River outlets were manually located for all basins that appear in the National Land Survey topographic maps. Finally, we extended coastal land cover classes [given by the second level classes of CORINE map (http://www.eea.europa.eu/publications/COR0-landcover)] offshore so that the shorelines and marine waters with less than 3 m of water depth (the analysis area) are assigned the land cover class of the adjacent land. CORINE land cover maps, with a minimum mapping unit of 25 ha, from years 2000 (for the Archipelago sites) and 2006 (for Svartbäck) were provided by SYKE.

Vector maps of *Phragmites* distributions were converted to binary grids of raster format indicating *Phragmites* presence and absence. Setting the grid size for conversion took into account the spatial resolution of the predictor grids. As the predictor grids in our case were derived and interpolated at grid sizes of 5 and 10 m, converting maps of *Phragmites* distributions to smaller grid size would result in bulky files with redundant information. However, the bigger the grid size used to convert vector maps to raster layers, the higher the loss of information on *Phragmites* distributions because small patches of *Phragmites* might disappear. In order to minimize the loss of information on *Phragmites* distribution, we computed the depth index, defined as the average distance from the polygon's interior point to the nearest point on the perimeter (Angel et al. 2010), for all *Phragmites* patches in each site. A maximum of one patch in each site was with a depth index less than 0.5 m. Therefore, *Phragmites* distribution maps were converted to raster grids of 2 m cell size, ensuring negligible loss of accuracy of *Phragmites* distribution. We then used a Latin Hypercube Sampling procedure (LHS) (Minasny and McBratney 2006; Falk et al. 2011) to draw samples of 10,000 and 100,000 instances from each site for different analysis purposes as explained below. LHS procedure samples variables from their multivariate distributions so that the range of each variable is fully covered by maximally stratifying its marginal distribution. In order to ensure representativeness, the samples were maximally stratified for *Phragmites* occurrence (presence/absence), each predictor and the geographic space (given by x and y coordinates).

### Analysis of *Phragmites* distribution

*Phragmites* spread into certain locations and its disappearance from others over years is a function of dispersion mechanism and location characteristics. While vegetative growth with rhizomes is said to be the major method by which *Phragmites* propagates (Bart and Hartman 2003; Gucker 2008), part of *Phragmites* dynamics is explained by seed germination and seedling growth (Alvarez et al. 2005; McCormick et al. 2010). When suitable conditions exist, reed colonies can establish on clear shores (settlement phase) and start propagating vegetatively (Koppitz and Kühl 2000). We analyzed changes in the areal cover of reed colonies between time periods in each site using the logarithmic growth equation given by Wilson and Bossert (1971; Rice et al. 2000). The equation is given as follows: *N = N*_*0*_ *e*^*rt*^, where *N*_*0*_ is the area of reed patches at time 0, *N* is the area of reed patches at time 1, *e* is the base of the natural logarithm, *t* is the difference in years between time 1 and time 0, and *r* is the intrinsic rate of natural increase per year, for which the equation is solved. This allows comparing figures of reed growth in different geographic areas over time because the formula normalizes change for different areas and time periods. In this section, we analyze the occurrence and spread of *Phragmites* in relation to environmental factors and neighborhood effect.

#### *Phragmites* occurrence and suitability factors

Four environmental variables were used to examine the habitat suitability for *Phragmites*. These variables are motivated by the review of *Phragmites* ecology presented in the Introduction. The first variable is the distance to the closest river outlet, which is used as a surrogate variable for nutrient content in sediments. Excessive amount of nutrients is washed out to rivers and transported to the land–sea interface. A fraction of river-transported matter is accumulated in the sediments, and further dispersed matter is diluted and mixed with the sea water as the distance offshore grows (Rodrigues et al. 2009), making shorelines and waters close to river outlets suitable for *Phragmites* spread. The second variable is the land cover given by CORINE maps. The third variable is the water depth, which is included in the predictors because it limits the ability of *Phragmites* to expand seaward (Huhta 2009; Meriste et al. 2012). Finally, shore openness is included in the predictors as an indication of wave exposure, an essential factor for *Phragmites* ability to colonize shores (Coops et al. 1996; von Numers 2011), and for the ecological structure of shoreline communities (Ekebom et al. 2003; Tolvanen and Suominen 2005).

We examined distributions of these variables for reed-occupied and reed-clear calls in the sites using the samples with 100,000 instances. Locations that hosted *Phragmites* in any year were considered reed-occupied, and those where *Phragmites* never existed (according to the data at hand) were labeled unoccupied. This allows exploring the occurrence of *Phragmites* at different ranges of the tested variables in different geographic areas. In order to examine the progression of *Phragmites* over time in relation to the variables, we created density plots for the reed-occupied cells in each year for every site. We note that the latter analysis included only the static predictors, namely the water depth, the relative openness, and the distance to river mouths; land cover is varying over time, and the data we have are from years 2000 and 2006, which is why it was not included in this analysis.

#### *Phragmites* expansion and neighborhood effect

Dispersal is a major cause of intrinsic SAC in ecology (Dormann 2007a). Species distribution is strongly influenced by the ability of propagules to reach suitable habitats. In clonal species such as *Phragmites*, vegetative growth leads to the occurrence of large clusters of colonies once established in a location. In an aggregative process, the state of the neighborhood is an important determinant of the future state of the location in question. In addition to the endogenous source of autocorrelation (*i.e.,* dispersal), an exogenous component of spatial dependency (Legendre et al. 2002) also exists as most environmental variables are autocorrelated. Therefore, the presence of *Phragmites* in a location can indicate the suitability of its surrounding (where similar conditions are likely to be found) for *Phragmites* takeover.

We used Svartbäck *Phragmites* data to analyze the influence of the neighborhood on the future of a location with respect to *Phragmites* presence/absence. The choice of this site was because of the short time lag (3 years) between the available *Phragmites* maps, which allows detecting the effect of the neighborhood composition on *Phragmites* progression, unlike the long time periods (13–20 years) separating maps of *Phragmites* in the Archipelago sites. We examined the neighborhood effect on multiple scale settings because the representation of phenomena and variables in the analysis environment can greatly influence the results (Higgins et al. 1996; Roddick and Lees 2009). Different sizes and shapes of cells and neighborhood analysis windows can result in confounding conclusions on the autocorrelation of variables. Analysis of the influence of neighbors beyond the first order is advised (Cliff and Ord 1969; cited in Fortin and Dale 2009), and an appropriate setting of the neighborhood window allows capturing the operational range of the process being modeled (White and Engelen 2000). We examined diameters of the maximum inscribed circle (Angel et al. 2010) in patches that emerged or disappeared in the period from 2003 to 2006 in order to determine the appropriate cell size for the analysis. The lower and upper quartiles were 2 and 10 m, based on which the analysis was conducted on *Phragmites* grids with cell sizes of 3, 5, 7, and 9 m. We also considered various sizes of neighborhood windows, including 3 × 3, 5 × 5, 7 × 7, and 9 × 9.

**Figure 3:**
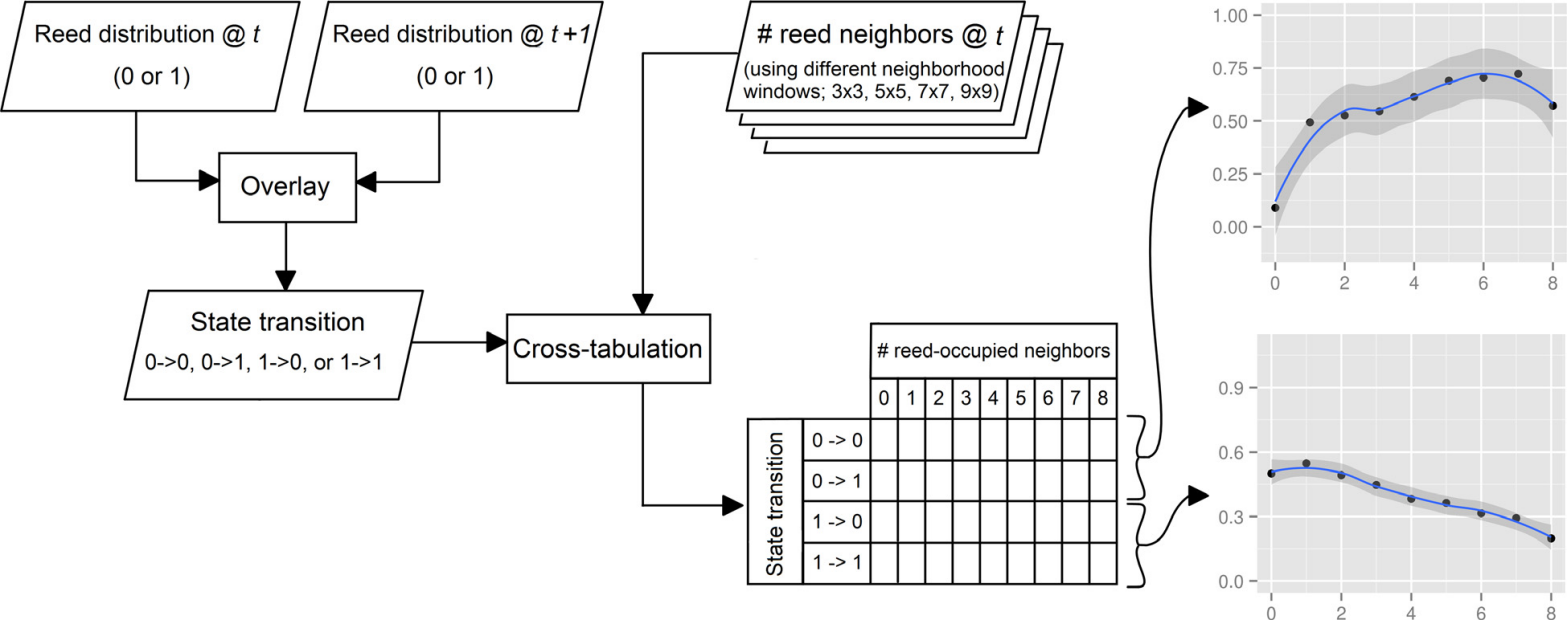
Diagram of neighborhood analysis. The analysis results in figures (on the right-hand side) of neighborhood effect on the dynamics of reed; *x*-axes give the number of reed-occupied cells within the neighborhood analysis window (in this example 3 × 3) in the initial time step, and *y*-axes give the proportion of cells where reed expanded (upper graph) or disappeared (lower graph) by the next time step.

The approach to exploring the neighborhood effect on *Phragmites* dynamics is illustrated in Fig. 3. Overlaying the boolean-valued *Phragmites* grids of 2003 and 2006 results in a grid representing the state transition of cells. This can be one of four possibilities; cells that were free of *Phragmites* in both time steps (denoted 0–>0), cells to which *Phragmites* had expanded by 2006 (denoted 0–>1), cells from which *Phragmites* had disappeared by 2006 (denoted 1–>0), and cells that were occupied by *Phragmites* in both time steps (denoted 1–>1). Another set of grids are those holding the number of reed-occupied neighbors for each cell in 2003, using different neighborhood windows. Those were, separately, cross-tabulated with the grid holding the state transition producing four two-way tables. Each table was then split based on the state of 2003 (0–>0 with 0–>1 and 1–>0 with 1–>1). A picture of the neighborhood effect can thus be depicted; the likelihood of a location to become occupied by *Phragmites* is given by the proportion of locations with the same neighborhood composition to which *Phragmites* had expanded, and the likelihood of *Phragmites* to disappear from a location is given by the proportion of locations with the same neighborhood composition from which *Phragmites* had disappeared.

### Modeling *Phragmites* distribution

Aiming at a predictive SDM, we adopted the boosted regression trees (BRT) method (De'ath 2007; Elith et al. 2008). Although BRT techniques stem from both statistical and ML approaches (Elith et al. 2008), the dependence on algorithmic learning, the focus on predictive accuracy, and the use of large datasets for learning characterize it as a ML approach (Hochachka et al. 2007; Olden et al. 2008). BRT comprises decision trees for classification and regression and boosting optimization for combining a collection of models (Elith et al. 2008). In a tree-based model, the predictor space is repeatedly divided into areas, using split points that minimize prediction errors. Each area is then assigned to the most probable class. Instead of building a single tree with best prediction, boosting optimizes accuracy (Ridgeway 1999; Sutton 2005) by gradually adding up trees that best reduce the loss in prediction performance. When training a BRT model, the bag fraction (*bf*), the learning rate (*lr*), and the tree complexity (*tc*) parameters should be set. The *bf* introduces stochasticity into BRT by specifying the proportion of training data to be selected at each step of the model building. The *lr* determines the contribution of each new added tree to the model deviance reduction, and the *tc* determines whether predictor interactions are to be fitted by defining the number of nodes in each tree. Although a slower *lr* and a higher number of trees are generally preferable (Elith et al. 2008), there is no concrete definition of best combinations of those parameters. For a comprehensive description of BRT particularly for ecological applications, the reader can refer to De'ath (2007), Elith et al. (2008), and Elith and Graham (2009).

We used the samples with 10,000 instances to build BRT models for each site with various combinations of *lr* (0.05, 0.01, 0.005, 0.001), *tc* (3, 5, 7, 10, 20), and *bf* (0.25, 0.50, 0.75, 0.90) parameters. A *bf* of 0.75, a *lr* of 0.005, and a *tc* of 10 almost consistently yielded higher performance than other combinations. We used these values of parameters to build models for *Phragmites* prediction. We used the extension of R *gbm* (Ridgeway 2006) for BRT developed by Elith et al. (2008) for building BRT models. We tested the models for both interpolation and extrapolation of *Phragmites* occurrences. For the interpolation, the sample drawn from each site was used to classify independent cells from the same site. As available CORINE land cover maps are from years 2000 and 2006, we used *Phragmites* distributions of 1996 in Ruissalo, 1995 in Kramppi, 1995 in Redamo, and 2006 in Svartbäck to build and test the models for interpolation. For the extrapolation, we used the model trained with the sample of Ruissalo 1996 to predict *Phragmites* distributions of 1995 in Kramppi, 1995 in Redamo, and 2003 in Svartbäck. The choice of Ruissalo model for extrapolation was because the land cover grid of Ruissalo is inclusive of all land cover classes that appear in other sites.

Models and predictions were evaluated using a number of measures (Pearce and Ferrier 2000). For BRT models, we present the mean and standard error of ten-fold cross-validation (CV) statistics including correlation and the area under the receiver operative characteristic (ROC) curve (AUC). AUC statistic was also calculated for the interpolated and extrapolated maps of *Phragmites* distribution using R *ROCR* (Sing et al. 2005). Probability estimates of *Phragmites* occurrence were dichotomized to binary data of presence/absence using the minimized difference threshold criteria (Jiménez-Valverde and Lobo 2007). Sensitivity and specificity performance measures were computed using a cell-by-cell comparison of predicted distributions with truth.

## Results

### Analysis of *Phragmites* distribution

#### Figures of *Phragmites* spread over years

Analysis of *Phragmites* distribution in different years in the study sites showed an increased *Phragmites* colonization of clear waters and shoreline segments. Various *Phragmites* prevalence (Table 1) and annual intrinsic rates of increase (Table 2) were observed in different geographic areas and time periods. Expansion of *Phragmites* was the dominant trend in most examined periods. An exception was in Kramppi between 1977 and 1995 where the area of reed colonies shrank with a small annual rate of 0.4%. However, over the whole period (1962–1995), Kramppi witnessed a significant growth of reed colonies. Highest reed prevalence and annual expansion rate occurred in Svartbäck where *Phragmites* covered one-fifth and one-fourth of the area in 2003 and 2006, respectively.

**Table 1:** Area and percentage of *Phragmites* in different years in comparison with the total analyzed area in each study site.

Site	Ruissalo			Kramppi			Redamo			Svartbäck	
Total area (ha)	427.16			170.51			259.30			1083.70	
Year	1962	1982	1996	1962	1977	1995	1963	1982	1995	2003	2006
*Phragmites* area (ha)	48.05 (11%)	59.78 (14%)	69.87 (16%)	2.58 (2%)	19.36 (11%)	17.86 (10%)	14.79 (6%)	15.05 (6%)	20.83 (8%)	226.73 (21%)	288.36 (27%)

**Table 2:** Intrinsic rate of increase in *Phragmites* areal cover per year in each study site over different periods, calculated using a logarithmic growth equation

Site	Overall period	Intrinsic rate of increase (year^−1^),%	In between periods	Intrinsic rate of increase (year^−1^),%
Ruissalo	1962–1996	1.1	1962–1982	1.1
			1982–1996	1.1
Kramppi	1962–1995	5.9	1962–1977	13.4
			1977–1995	−0.4
Redamo	1963–1995	1.1	1963–1982	0.1
			1982–1995	2.5
Svartbäck	2003–2006	8.0		

#### Relationships between *Phragmites* occurrence and predictor variables

**Figure 4:**
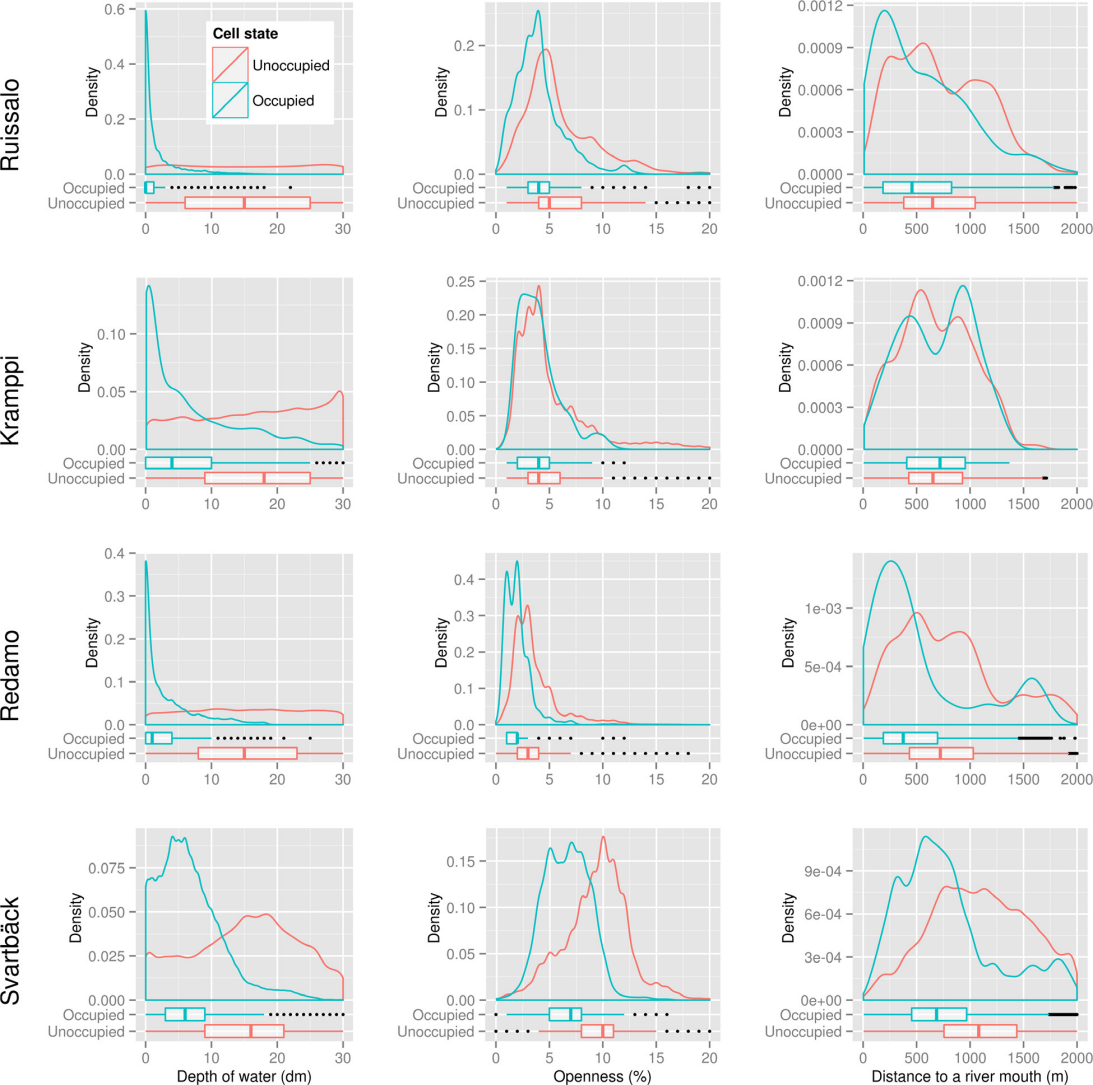
Variable distribution for reed-occupied cells versus clear cells in each study site.

**Figure 5:**
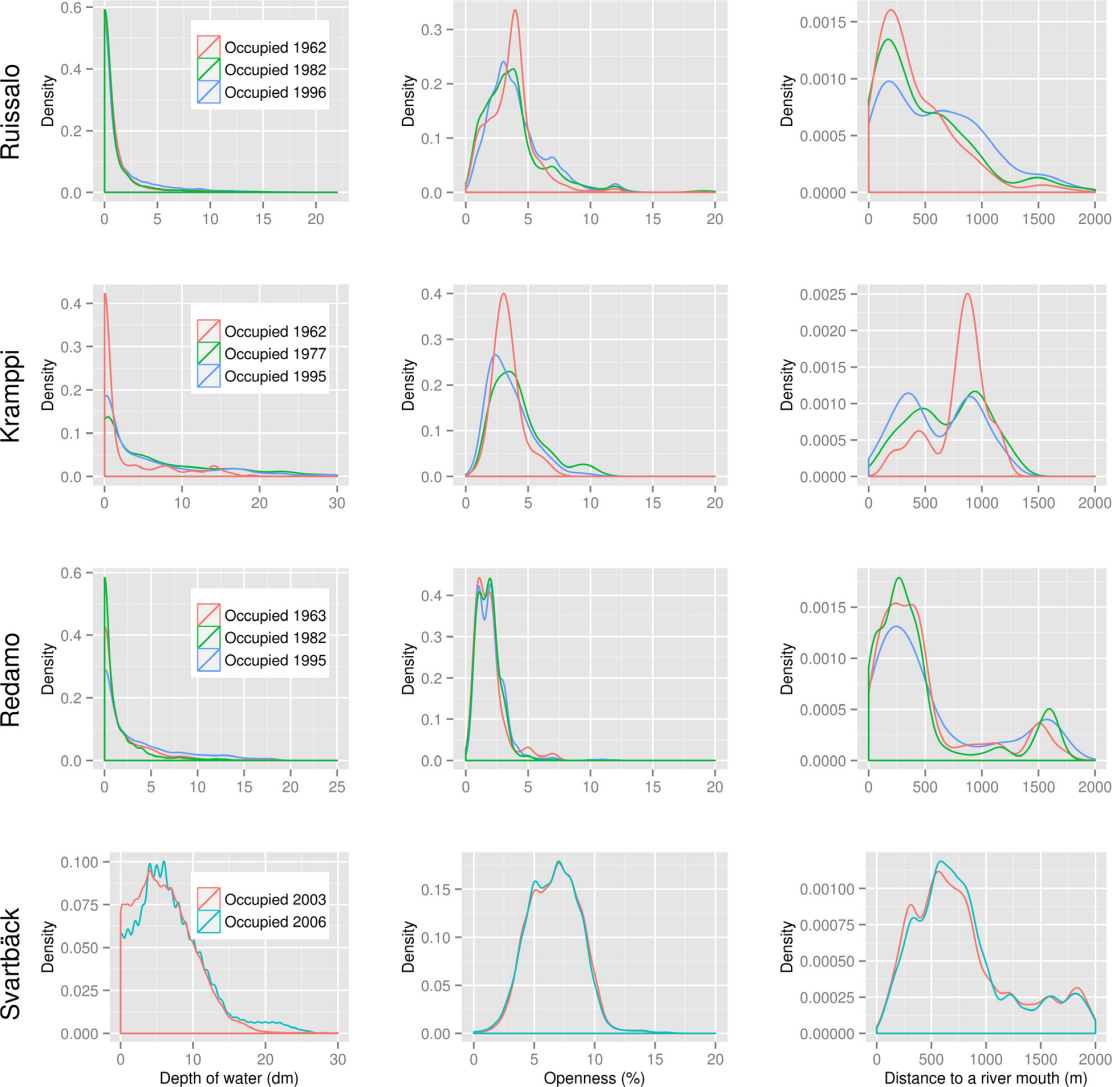
Variable distribution for reed-occupied cells in different years in each study site.

**Figure 6:**
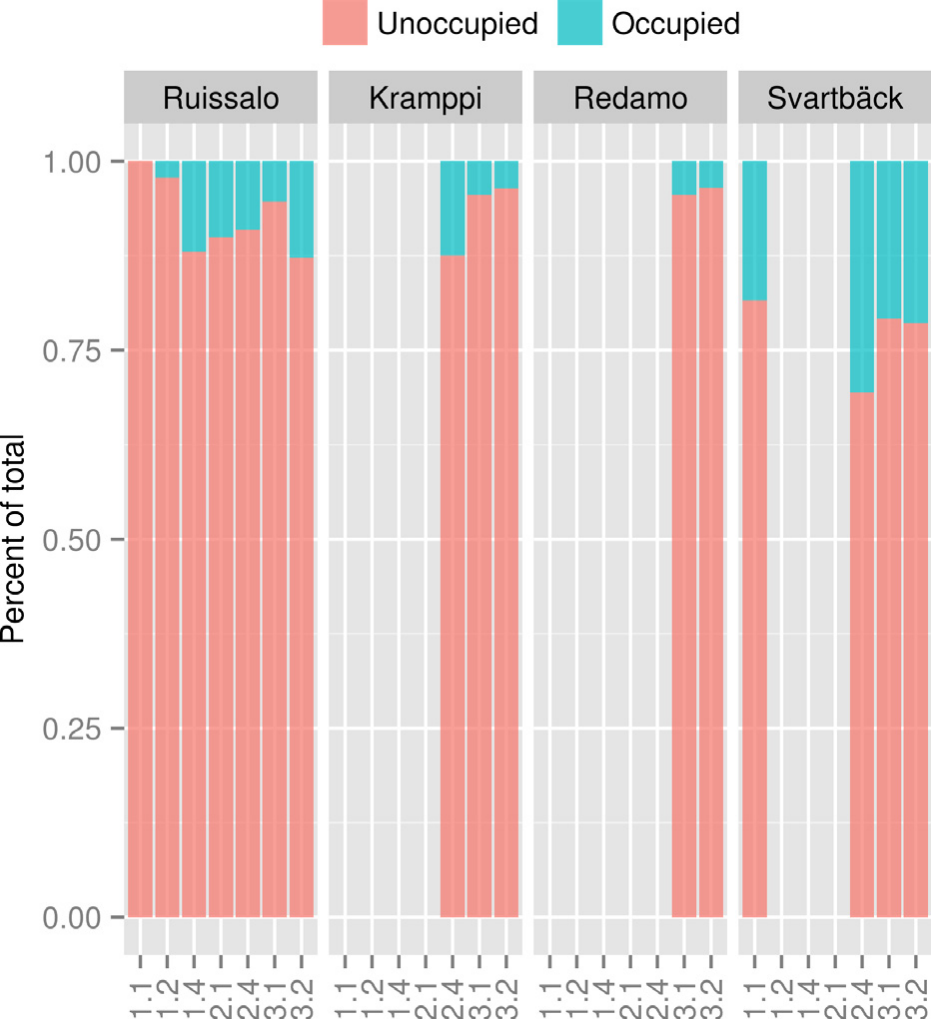
Prevalence of *Phragmites* on the shoreline of different land covers analyzed using (i) CORINE 2000 land cover map and *Phragmites* distrintuion of 1996 in Ruissalo and 1995 in Kramppi and Redamo, and (ii) CORINE 2006 land cover map and *Phragmites* distrintuion of 2006 in Svartbäck. Codes are according to CORINE nomenclature; 1.1 Urban fabric; 1.2 Industrial, commercial, and transport units; 1.4 Artificial, nonagricultural vegetated areas; 2.1 Arable land; 2.4 Heterogeneous agricultural areas; 3.1 Forests; 3.2 Scrub and/or herbaceous vegetation associations.

Differences were observed in *Phragmites* prevalence in relation to the same variable in different geographic areas (Fig. 4) and, to a lesser extent, in different time periods (Fig. 5). Notable prevalence of reed is found on the shorelines and shallow waters. Majority (3 quartiles) of the reed colonies in all examined sites existed in waters with less than 1 m in depth. However, the distribution of *Phragmites* over time shows progression into deeper waters, especially in Kramppi and Svartbäck. *Phragmites* also dominated sheltered shorelines and bays, although on the temporal scale seemed to have expanded to slightly less sheltered areas, especially in Ruissalo and Kramppi. Areas in the vicinity of river outlets are also observed to provide suitable habitats for *Phragmites*. It is worth noting the distance lag from river outlets before *Phragmites* occurs in high prevalence. Areas right at the outlets of river basins show lower or no suitability for *Phragmites* compared to those within proximity of 250 m (in Ruissalo and Redamo) to 600 m (in Svartbäck). *Phragmites* appears to colonize shores adjacent to various land cover types (Fig. 6) with slightly varying prevalence. Most sites had low representation of land cover classes. Heterogeneous agricultural areas (land cove class 2.4) seem to have high prevalence of *Phragmites* on their shores. *Phragmites* prevalence exhibited strong association with artificial, nonagricultural vegetated areas (class 1.4), and the scrub and herbaceous vegetation (class 3.2) in Ruissalo.

#### Neighborhood effect on *Phragmites* expansion

**Figure 7:**
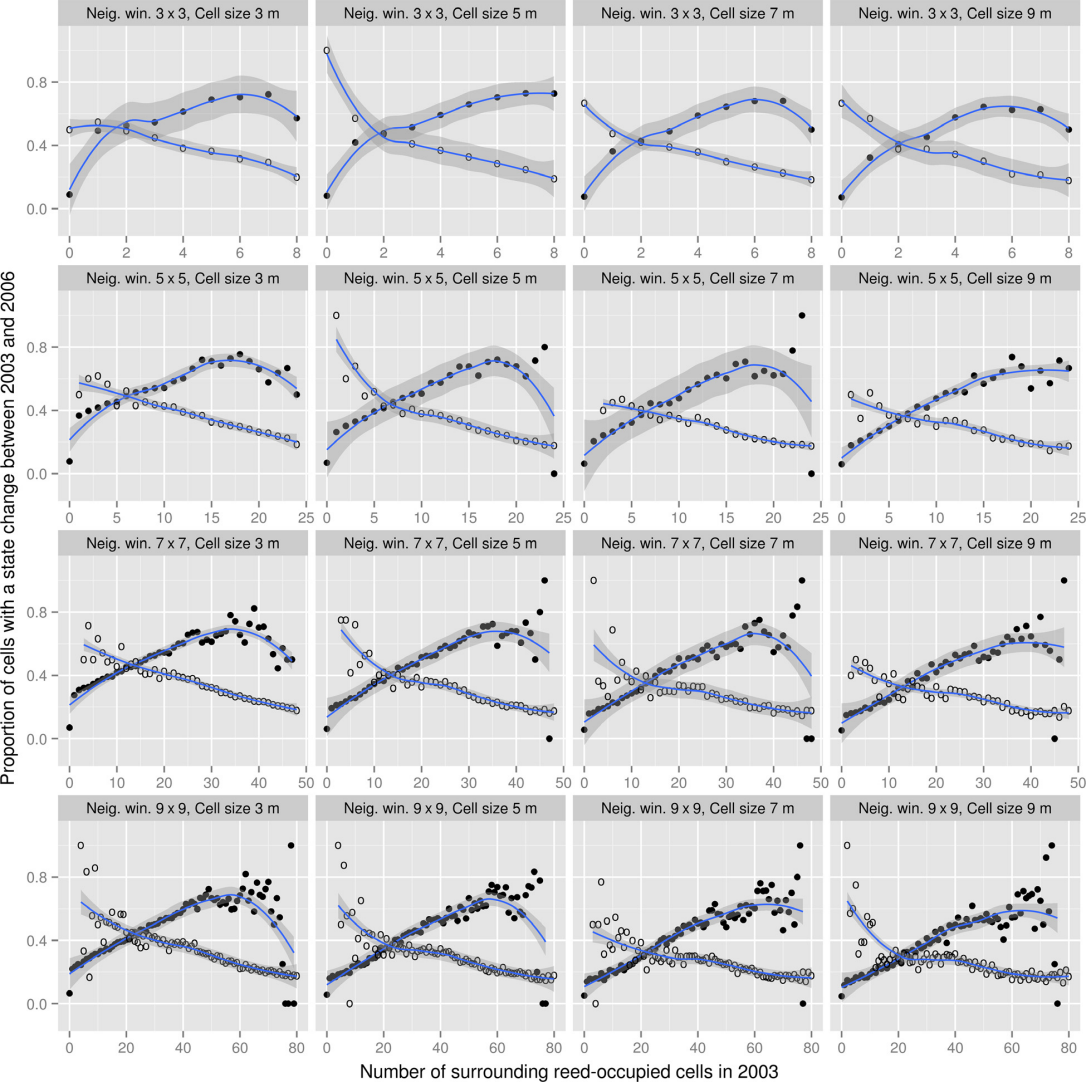
Influence of the neighborhood composition on the likelihood of a location to become occupied by *Phragmites* (solid circles) or unoccupied (open circles), analyzed using different cell sizes and neighborhood windows. The relationship illustrated by solid circles reflects the probability of a clear location to become colonized by *Phragmites* in the next years (state transition: 0–>1), while the relationship illustrated by open circles reflects the probability of reed to disappear from a location in the next years (state transition: 1–>0), given the composition of the location's neighborhood (count of reed-occupied neighbors).

Effect of the neighborhood composition on the transition of cells is illustrated in Fig. 7. The neighborhood composition, given by the number of reed-occupied cells surrounding the cell in question, is found influential on the likelihood of *Phragmites* to spread or disappear from a location. Generally, clear water cells with prevalence of *Phragmites* in the surrounding are more susceptible for *Phragmites* takeover. On the contrary, the likelihood of disappearance is higher for small standalone patches than it for patches surrounded by large *Phragmites* colonies.

While the same general picture of neighborhood effect is reflected in all combinations of cell sizes and neighborhood windows analyzed, differences can be noticed in the level of information and noise in curves from different combinations. Considering the 0–>1 state transition (the solid circles in Fig. 7), a small cell size (3 m), and a small neighborhood window (3 × 3) yielded no clear distinction in the probability of a cell to become colonized by *Phragmites* when 1 or 8 neighbors are already colonized. Enlarging the cell size and including neighbors beyond Moore neighborhood in the analysis gradually reflects a clearer picture of the neighborhood influence. However, when both the cell size and the neighborhood window are very large (9 m and 9 × 9), noise is introduced in the relationship curves, especially at high neighbor counts. Relationships for the 1–>0 state transition (the open circles in Fig. 7) reflected a trend of *Phragmites* disappearing when few adjacent colonies occur within the vicinity. When the neighborhood window is extended (7 × 7 and 9 × 9), noise is introduced at low neighbor counts.

### Modeling *Phragmites* distribution

**Figure 8:**
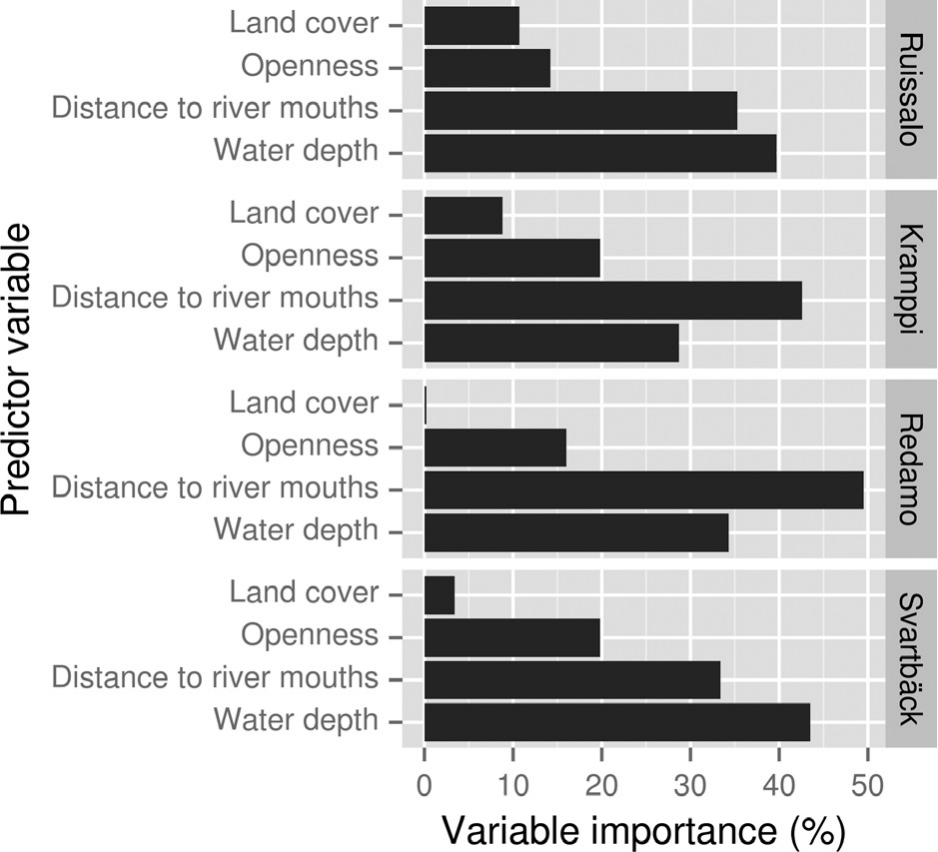
The influence of variables (in a scale of 100) on the prediction of *Phragmites* presence/absence in BRT models of each study site.

BRT models performed differently in predicting *Phragmites* distributions in different geographic areas. AUC scores from model CV were relatively high with values of 0.97 for Ruissalo and Redamo, 0.96 for Kramppi, and 0.89 for Svartbäck models. Table 3 lists the number of trees and statistics of model performance using CV. The influence of variables on the prediction of *Phragmites* occurrence is illustrated in Fig. 8 for each site. The depth of water (in Ruissalo and Svartbäck) and the distance to river mouths (in Kramppi and Redamo) were the most influential variables in predicting reed occurrences. Openness came third in variable importance in all sites, followed by the land cover, which was in some cases of negligible influence on the prediction.

**Table 3:** Number of trees and evaluation statistics of BRT models trained for each site. Mean and standard error values of correlation and AUC are calculated from ten-fold cross-validation.

Site	Number of trees	Mean correlation (se)	Mean AUC (se)
Ruissalo	5300	0.695 (0.016)	0.966 (0.004)
Kramppi	5800	0.665 (0.009)	0.956 (0.005)
Redamo	4100	0.645 (0.013)	0.966 (0.003)
Svartbäck	6650	0.616 (0.006)	0.886 (0.003)

**Figure 9:**
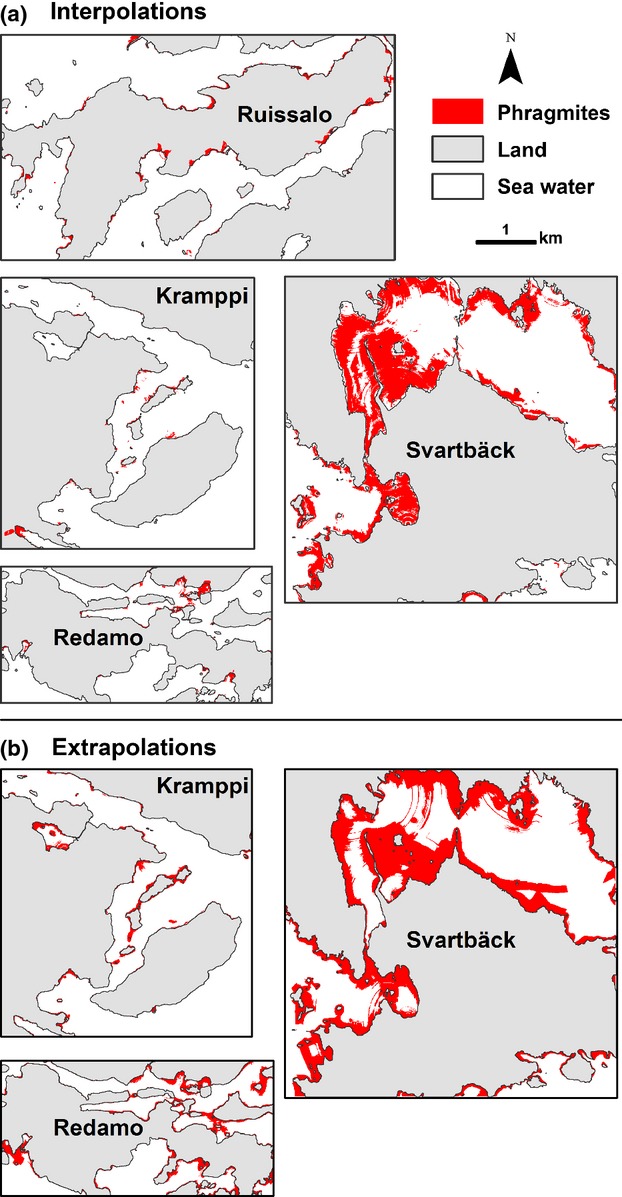
Predicted reed distribution in each study site. (a) Interpolated *Phragmites* distributions of Ruissalo 1996, Kramppi 1995, Redamo 1995 and Svartbäck 2006 from models trained with data from the site itself. (b) Extrapolated *Phragmites* distributions of Kramppi 1995, Redamo 1995 and Svartbäck 2003 from Ruissalo 1996 model. Probability estimates in both were dichotomized to show *Phragmites* occurrence using minimized difference threshold criteria.

*Phragmites* predictions from interpolations and extrapolations are shown in Fig. 9. AUC, sensitivity and specificity of predictions are listed in Table 4. All models performed well in the interpolation task, with higher performance in the Archipelago sites (AUC > 0.96) compared with Svartbäck (AUC = 0.89). Extrapolating the model trained with data from Ruissalo to other sites yielded lower, yet acceptable accuracy. AUC from model extrapolation compared with truth was 0.81 in Kramppi, 0.85 in Redamo, and 0.75 in Svartbäck.

**Table 4:** Evaluation of model performance in interpolating and extrapolating *Phragmites* distributions through a cell-by-cell comparison of resultant suitability maps with truth.

Site of training	Site of prediction	AUC	Sensitivity	Specificity
Ruissalo	Ruissalo	0.970	0.875	0.929
Kramppi	Kramppi	0.962	0.805	0.941
Redamo	Redamo	0.970	0.838	0.944
Svartbäck	Svartbäck	0.886	0.762	0.824
Ruissalo	Kramppi	0.807	0.707	0.725
Ruissalo	Redamo	0.847	0.870	0.695
Ruissalo	Svartbäck	0.753	0.762	0.637

## Discussion

The intrinsic rate of increase in *Phragmites* areal cover varied between study sites. The intensive progression of *Phragmites* in Svartbäck indicates the existence of highly suitable conditions for *Phragmites* proliferation. This is in line with figures showing that nutrient concentrations increase eastward in the GOF (SYKE 2009). While the increase in area was the observed trend, decrease in *Phragmites* areal cover and disappearance of reed patches occurred in some locations, most notably in Kramppi in the period 1977–1995. Those patches were mostly small in area and were not surrounded by other settlements of *Phragmites*. Increased *Phragmites* area eightfold in Kramppi, most of the reed patches that appear in the map of 1977 were not observed in the map of 1962. In the period from 1977 to 1995, most patches appeared in both maps with different areas (Fig. 2). This may suggest that changes in the reed distribution in Kramppi occurred by seed/seedling establishment in earlier years and by vegetative spread with rhizomes in following years.

The predictors used were all proxies to more functionally relevant variables, resulting in models for prediction rather than explanation. Predictors exhibited different levels of separation between the reed-occupied and clear locations. In Kramppi, for instance, the distributions of reed-occupied and clear cells almost overlap with respect to the openness and the distance to river mouths. We investigated this on a topographic map of Kramppi and found that it might be due to the presence of a relatively close bay (called Vanhankylänlahti) to which a number of rivers flow with no records of *Phragmites* occurrence in our data although recent Satellite Imagery from Google Maps (http://www.maps.google.com) indicates the presence of large reed colonies in that bay. This is likely what caused the two populations (reed-occupied and reed-free cells) to exhibit large overlap in their distributions. Based on this observation and given the good performance of the models, we argue that these variables have high potential in predicting *Phragmites* occurrences.

The suggested occurrence of *Phragmites* in locations where water depth exceeds 2 m in Fig. 4 should be treated critically as literature and personal observations indicate that it is unlikely for *Phragmites* to progress into waters deeper than 2 m (Luther 1951; Munsterhjelm 1997; L. Nurminen, pers. obs.). As no ground truthing was conducted to validate these particular data records, they should be regarded erroneous, and no conclusions about the limit of water depth at which *Phragmites* can occur should be drawn on their basis. Possible error sources that may have caused this are (i) errors in the interpolated bathymetry model, (ii) errors in the input maps of reed due to possible difficulties in distinguishing *Phragmites* on the aerial photographs, and/or (iii) errors due to the resolution inconsistency of *Phragmites* grids (2 m) and the bathymetry grid (5 m). It should be noted, however, that such possible errors in data have minimum effect on the results of BRT models due to their robustness to noisy data, as illustrated by the low error rates of the predictions. The use of binomial deviance, rather than AdaBoost, as BRT loss functions is likely to yield better performance where classes may be mislabeled (Elith et al. 2008), as the case may be here.

Similar to results from the exploratory analysis (Fig. 6), fitted values from the BRT model for *Phragmites* presence next to different land cover types (Fig. 10) show influence of agricultural areas in Ruissalo on the prevalence of *Phragmites*. In BRT models, the land cover was found to be the least influential of all variables in predicting reed occurrences. This may be due to the low resolution of the land cover grid, which was not detailed enough to (1) depict changes in the prevalence of *Phragmites* in areas adjacent to different classes of land cover or (2) include a wider range of land cover classes. Nonetheless, land cover types have diverse effects on ecological niches on the regional scale (Dormann 2007b; Hirzel and Le Lay 2008).

**Figure:**
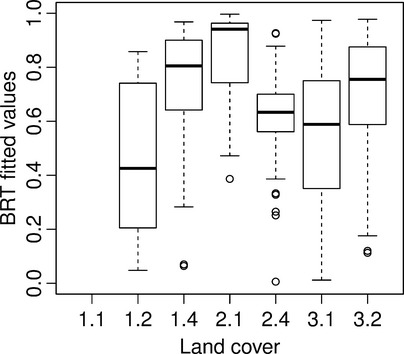
Fitted values from Ruissalo BRT model showing the influence of different land cover types on the prediction of *Phragmites* presence. Codes of land cover classes are according to CORINE nomenclature; 1.1 Urban fabric; 1.2 Industrial, commercial, and transport units; 1.4 Artificial, nonagricultural vegetated areas; 2.1 Arable land; 2.4 Heterogeneous agricultural areas; 3.1 Forests; 3.2 Scrub and/or herbaceous vegetation associations.

Refining the predictor variables and adding additional relevant predictors may enhance prediction and model performance. Some studies have suggested that land use and nutrient loading influence the prevalence of *Phragmites* on the local scale (Bertness et al. 2002; Silliman and Bertness 2004), while other studies have found their effect to be on the catchment scale (King et al. 2007; Kettenring et al. 2010). Therefore, improvement of the model results could be achieved by accounting for the river basin size and adjacent land use in order to define the magnitude and range of their impact on coastal areas. Also, more detailed bathymetry and openness models could result in a more accurate spatial prediction of areas suitable for *Phragmites*. Finally, seabed type in shallow waters can be a good indicator of a location's suitability for *Phragmites* (Coops and van der Velde 1995; Coops et al. 1996; Kaitaranta et al. 2013).

Neighborhood state was found influential in *Phragmites* dynamics. With vegetative proliferation, *Phragmites* is likely to overtake areas adjacent to existing colonies, provided favorable conditions exist (Koppitz and Kühl 2000). Considering cells beyond Moore neighborhood (the eight nearest cells) was useful for capturing the neighborhood influence on the expansion and disappearance of reed. However, very large neighborhood windows yielded trends with noise at high (in the 0–>1 case) and low (in the 1–>0 case) occupied-neighbor counts. We expect this to be a result of a strong clustering in *Phragmites* distribution in space which makes it unlikely to find many small patches of *Phragmites* standing alone in open waters or many spots of clear water in the middle of large reed colonies. Therefore, with few records for these cases, no clear trend could be depicted. Regardless of the settings chosen in this study, however, high likelihood of *Phragmites* spread into adjacent locations was found when significant prevalence of *Phragmites* occurs in the neighborhood.

Using a cellular data model in this type of studies has a number of advantages. It is compatible with the raster format widely used in GIS, which makes a range of free and open source software and libraries available for the computation and data manipulation. We used R *gbm* package (Ridgeway 2006; Elith et al. 2008) for modeling *Phragmites* distributions, R *ggplot2* (Wickham 2009) for illustrations, and Python Numpy (http://www.numpy.org/) for data manipulation and per-processing. The computational power of Numpy allows efficient development and running of dynamic models such as the cellular automata for spatial processes (Altartouri and Jolma 2012). From an ecological point of view, this data model is suitable for incorporating mechanisms of dispersal in SDM. The flexibility in setting the size and shape of the neighborhood window allows accounting for both close-range and long-distance dispersion. However, the ability to correctly model such processes is conditioned on the adequate choice of scale parameters such as the cell size and neighborhood window. Examining a range of settings for these parameters is important for choosing an adequate scale that maintains balance between model accuracy and generality. A disadvantage of this data model arises from the necessary conversion between vector and raster formats, as species distribution data are usually collected in a point or a polygon vector format. This may lead to accuracy reduction during the conversion. However, the loss of information can be minimized if landscape metrics and proper sampling techniques are considered in the conversion process.

*Phragmites* distribution was successfully modeled using BRT, with variation in the performance between the interpolation and extrapolation, and in extrapolating to different time periods and geographic locations. For the interpolation cases, BRT models for the Archipelago sites performed highly with AUC greater than 0.96. Lower but acceptable performance was observed in Svartbäck with an AUC of 0.89. This can be due to the presence of variables influencing *Phragmites* distribution in this site that were not included in our predictors. Also, we notice disappearance of *Phragmites* next to the shoreline in Svartbäck in 2006 compared with 2003, which might be due to manual removal of reeds, a factor not incorporated in the model. Nonetheless, our results concur with earlier work (Caruana and Niculescu-Mizil 2006; Elith et al. 2006, 2008; Elith and Graham 2009), suggesting the high potential of BRT in modeling species distributions.

*Phragmites* distributions were extrapolated on the spatial and temporal axes of model generalization (Hirzel and Le Lay 2008). Extrapolation of Ruissalo model to predict *Phragmites* distributions of Kramppi and Redamo yielded better performance compared with its performance in Svartbäck. Due to their spatial and temporal proximity to Ruissalo, conditions in the Archipelago sites (in the extrapolated years) are expected to be more similar than those in Svartbäck located in the Eastern part of the GOF. Extrapolation is usually burdened with uncertainty (Dormann 2007b; Elith and Leathwick 2009) and lower prediction performance in this case can be attributed to differences in the influential factors and their magnitude of influence in different sites. While the extrapolation of Ruissalo model in other sites demonstrates the potential of the methods presented in this study for predictive mapping of *Phragmites*, the direct application of the model along the whole Finnish coast is not suggested. For such task, we recommend zonation of the area into smaller areas with comparable characteristics, *for example* water quality figures, and training a model of each individual area in order to achieve accurate predictions.

Adequate interpretation of SDM results is important in order to avoid misuse (Keating and Cherry 2004; Jiménez-Valverde et al. 2008). Our study is correlative and employs a ML approach, which emphasizes prediction and utilizes any predictor that is potentially informative (Hochachka et al. 2007). The correct interpretation of the resulting maps is not an absolute probability of *Phragmites* occurrence but rather a relative ranking of habitat suitability (Keating and Cherry 2004; Morisette et al. 2006). Using datasets relatively easy to obtain, our study can help delineating suitable habitats for *Phragmites* along the Southern Finnish coasts, allowing early management plans to be made (Bart et al. 2006; King et al. 2007). Predicting habitat suitability is essential also for ecological studies on species distribution and habitat diversity (Lappalainen et al. 2008; Pitkänen et al. 2013) and timely predictions on the catchment-borne nutrient loading of coastal areas (Kaitaranta et al. 2013), given the key role of reed beds on littoral communities in shallow and sheltered coastal ecosystems (Meriste et al. 2012).

In conclusion, the dynamics of *Phragmites* at the Southern coastal zone of Finland has shown both expansion and disappearance of local patches, but clearly expansive growth has been the dominant trend, observed also by other studies from the Northern Baltic area (von Numers 2011; Meriste et al. 2012). Showing variation in different geographic locations, the progression of *Phragmites* resulted in an increase in the colonies areal cover by more than 1% per year in all analyzed sites, reaching 8% in some sites. The depth of water, shore openness, and proximity to river mouths were useful predictors of *Phragmites* occurrence. Our results indicate that shallow shores located nearby river outlets represent suitable habitats for *Phragmites* establishment and expansion. In concordance with von Numers (2011), over the last few decades, *Phragmites* has shown progression into slightly deeper waters and relatively more open shores. Although in the Baltic Sea, the potential seaward expansion of *Phragmites* is ultimately regulated by sea-level fluctuation and wave action reflecting the windiness and storminess of the sea area (Meriste et al. 2012). We also found the state of a location's surrounding in terms of *Phragmites* occurrence to influence the likelihood of *Phragmites* progression to that location. The resulting habitat suitability maps suggest the existence of places potentially suitable for *Phragmites* colonization. Extensive research in North America (Lambertini et al. 2008; Belzile et al. 2010) and Central Europe (Koppitz 1999; Koppitz and Kühl 2000; Fér and Hroudová 2009) has shown *Phragmites* to expand and colonize new areas mainly through close-range vegetative growth and long-distance generative dispersal by seeds. Nevertheless, close-range seedling dispersal may occur when seedling establishment is enabled, for example, by dredging or by settlement of organic matter on sandy shores through eutrophication, and, on the other hand, long-distant vegetative dispersal is enabled by detached rhizome bits of old stands (Fér and Hroudová 2009). To reveal the expansion dynamics of *Phragmites* in more detail in the Northern Baltic and the coastal area of Southern Finland, a detailed study on the population structure, genetic diversity, and reproduction mode of the reed stands would be timely and provide important supplementing information for the modeling approach.
